# Diagnostic Evaluation and Surgical Management of Self-Inflicted Trans-Frontal Sinus Penetrating Brain Injury in a Patient with Schizophrenia

**DOI:** 10.3390/diagnostics16152403

**Published:** 2026-07-30

**Authors:** Hak Sung Kim, Jae Ho Kim, Eun Ju Yoon, Sangwoo Ha

**Affiliations:** 1 Department of Neurosurgery, Chosun University Hospital, Chosun University College of Medicine, Gwangju 61453, Republic of Korea; loverei82@gmail.com (H.S.K.); rubg2day@gmail.com (J.H.K.); 2 Department of Radiology, Chosun University Hospital, Chosun University College of Medicine, Gwangju 61453, Republic of Korea; radyej@chosun.ac.kr

**Keywords:** diagnostic imaging, digital subtraction angiography, penetrating traumatic brain injury, frontal sinus, retained foreign body, *Serratia odorifera*

## Abstract

**Background:** Non-missile penetrating traumatic brain injury (pTBI) is a rare but life-threatening condition. In self-inflicted cases involving the frontal sinus, a rigorous preoperative diagnostic workup is crucial to assess foreign body fragmentation, trajectory, and potential vascular compromise. The complexities of such cases demand a highly structured approach to prevent severe secondary brain injury and long-term infectious sequelae. **Case Presentation:** A 36-year-old male with schizophrenia presented after attempting suicide by stabbing a kitchen knife through his frontal sinus. Multimodal diagnostic imaging was immediately employed. Skull radiographs and computed tomography (CT) precisely delineated the blade traversing the frontal sinus and entering the anterior cranial fossa. Crucially, given the proximity to the skull base, digital subtraction angiography (DSA) was proactively performed, which confirmed the absence of major cerebrovascular injury and safely guided the surgical strategy. Based on these precise imaging findings, a bifrontal craniotomy was performed. During the procedure, the main blade was extracted, and a retained broken metallic tip—recognized upon close intraoperative inspection of the extracted blade—was successfully retrieved from the intracranial compartment. Postoperative wound infections (with causative pathogens isolated as *Serratia odorifera* and coagulase-negative *Staphylococci*) were effectively eradicated with targeted antibiotics. The patient achieved a favorable neurological recovery. **Conclusions:** Multimodal neuroimaging, particularly the combination of CT and DSA, can be highly beneficial in the diagnostic workup of complex pTBI. Precise preoperative imaging helps guide the optimal surgical approach, ensuring complete foreign body removal and minimizing severe secondary complications. Early intervention, guided by standardized imaging and followed by aggressive medical management, can yield positive outcomes even in severely morbid presentations.

## 1. Introduction

Penetrating traumatic brain injury (pTBI) is a severe form of head trauma that presents significant diagnostic and therapeutic challenges to neurosurgical teams. While missile injuries, such as gunshot wounds, are more common and typically transfer a massive amount of kinetic energy leading to diffuse cavitational tissue destruction, non-missile penetrating injuries caused by sharp objects are rare and distinct. These low-velocity non-missile injuries can cause significant focal damage strictly along the anatomical trajectory of the offending weapon, often sparing adjacent tissue if major vascular structures are avoided.

Self-inflicted pTBI associated with underlying psychiatric disorders poses unique anatomical and surgical risks. When the trajectory involves the frontal sinus, the trans-sinus route breaches the barrier between the external environment, paranasal cavities, and the sterile intracranial space, elevating the risk of ascending infections such as meningitis. Furthermore, proximity to the anterior skull base and cerebral vasculature introduces potential risks of vascular injury. According to updated penetrating TBI guidelines, comprehensive diagnostic imaging is indicated to delineate the trajectory, evaluate vascular compromise, and detect retained foreign body fragments prior to surgical extraction, particularly when the weapon lies near major vessels [1,2]. In this report, we describe the multidisciplinary management of a complex self-inflicted trans-frontal sinus pTBI in a patient with chronic schizophrenia. We highlight the critical clinical lessons from this case, specifically focusing on the utility of digital subtraction angiography when CT angiography is obscured by metallic artifacts, the intraoperative recognition and retrieval of a retained blade tip, successful frontal sinus cranialization, and culture-directed antibiotic management of postoperative infection.

## 2. Case Report

**Figure 1:**
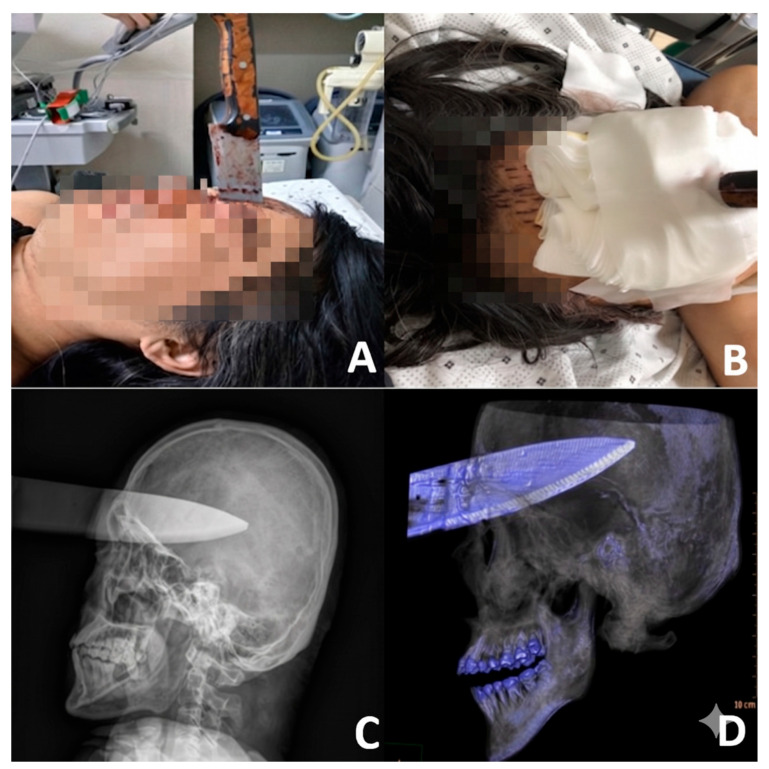
Preoperative clinical and radiographic findings. (**A**) Clinical photograph showing the vertical penetration of a kitchen knife in the midline glabellar region. (**B**) Superior view of the frontal scalp revealing multiple superficial “hesitation marks,” indicating failed attempts to penetrate the calvarium before the successful trans-sinus entry. (**C**) Lateral skull radiograph clearly demonstrating the angle and depth of the blade traversing the frontal sinus. (**D**) Sagittal view of the brain computed tomography (CT) scan (bone window) revealing the trajectory of the knife passing through the frontal sinus and entering the anterior cranial fossa.

A 36-year-old male presenting with a stuporous mental state following a severe suicide attempt was brought to our emergency department at 10:00 AM by emergency medical services. He had been discovered at approximately 09:00 AM by his mother at home with a kitchen knife embedded in his forehead. Upon arrival, his vital signs were stable (blood pressure 130/80 mmHg, heart rate 82 beats/min, body temperature 36.6 °C). Upon initial assessment in the trauma bay, the patient exhibited a Glasgow Coma Scale (GCS) score of 9 (E3V1M5). Pupil examination revealed equal and prompt light reflexes bilaterally (3 mm/3 mm) without focal lateralizing signs. Gross examination revealed a large, metallic kitchen knife vertically embedded deep in the midline glabellar region of the patient’s face (Figure 1A). A closer inspection of the surrounding anatomy revealed multiple superficial puncture wounds on the adjacent frontal scalp. These lacerations indicated “hesitation marks,” suggesting prior attempts where the patient had failed to penetrate the thick, rigid calvarium before successfully breaching the structurally weaker frontal sinus (Figure 1B).

A review of the patient’s medical and psychiatric history revealed that he had been managing chronic schizophrenia for seven years. His psychiatric condition was reportedly being treated with a complex pharmacological regimen that included risperidone, clonazepam, zolpidem, and carbamazepine.

To assess the exact nature and extent of the intracranial injury, a comprehensive sequence of diagnostic imaging was immediately performed. Initially, a lateral skull radiograph was obtained, which clearly depicted the dense metallic blade passing directly through the anterior and posterior tables of the frontal sinus (Figure 1C). At 11:00 AM (1 h post-arrival), a brain computed tomography angiography (CTA) scan was attempted as the initial screening modality. The CTA confirmed the exact trajectory of the weapon, demonstrating that the blade extended deep into the frontal lobe parenchyma (Figure 1D). However, evaluation of adjacent intracranial vasculature was severely limited by massive metallic streak artifacts caused by the dense metallic composition of the knife.

Crucially, given the anatomical trajectory’s close proximity to the floor of the anterior cranial fossa and the anterior cerebral artery territory, there was a high index of suspicion for traumatic vascular injury. It was strongly considered that any potential movement or manipulation of the blade during surgical extraction could precipitate catastrophic, uncontrollable hemorrhage if an underlying vascular laceration or pseudoaneurysm was present but obscured on the CTA.

**Figure 2:**
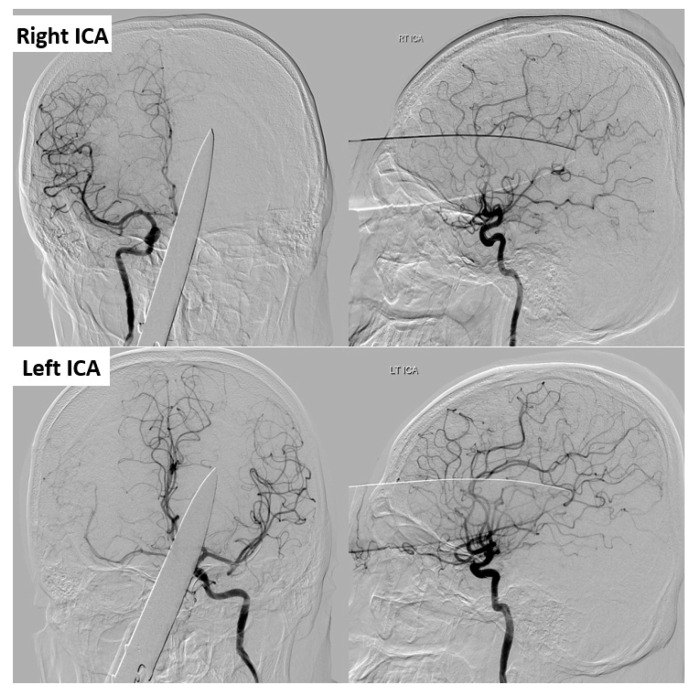
Preoperative vascular assessment. Digital subtraction angiography (DSA) of both internal carotid arteries demonstrating no evidence of traumatic aneurysm or major vascular injury, despite the close proximity of the foreign body to the anterior cerebral artery complex.

To definitively evaluate the integrity of the cerebral vasculature, digital subtraction angiography (DSA) was performed at 13:00 (3 h post-arrival) prior to foreign body extraction. The angiographic protocol comprehensively included selective bilateral internal carotid artery (ICA) injections with three-dimensional (3D) rotational angiography, as well as a left vertebral artery injection. External carotid artery injections were omitted as the relevant anterior circulation was clearly and sufficiently visualized. The dynamic angiographic runs ruled out any injury to the major arterial trunks, confirming the absence of traumatic pseudoaneurysms or active contrast extravasation (Figure 2). Given the definitive negative findings on the initial comprehensive DSA and the patient’s stable clinical course without signs of delayed hemorrhage, routine repeat vascular imaging was not performed postoperatively.

Following the meticulous diagnostic workup, emergency surgical intervention was initiated at 16:00 (6 h post-arrival). Given the midline nature of the lesion, a bifrontal approach was selected. The patient was placed in a supine position with Mayfield pin fixation, and the neck was slightly extended to position the orbital rim as the highest point. An approximately 10 cm bifrontal bone flap was elevated across the midline. During the exposure, the anterior portion of the superior sagittal sinus was found to be rudimentary; therefore, additional interventions such as venous ligation were not required. This wide surgical approach was designed to allow for the safe, direct, and controlled removal of the retained kitchen knife under direct microsurgical visualization. The main body of the knife was extracted carefully along its axis of entry.

**Figure 3:**
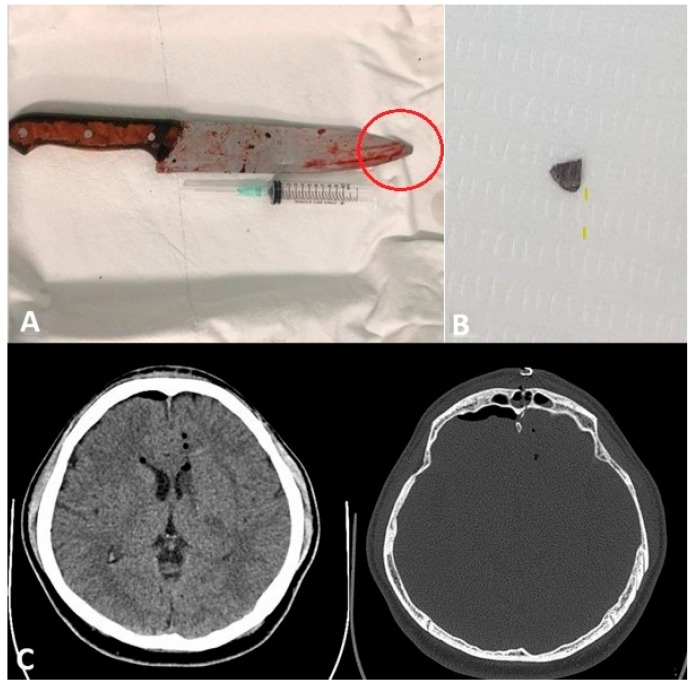
Intraoperative and postoperative outcomes. (**A**) Photograph of the extracted kitchen knife (shown with a 10 cc syringe for scale). Note the blunted tip suggesting fragmentation (red circle). (**B**) The recovered metallic tip fragment (approximately 7 mm in length) which was found embedded near the inner wall of the frontal sinus and successfully removed. (**C**) Postoperative axial CT scan showing total removal of the foreign body and cranialization of the frontal sinus.

However, a close intraoperative inspection of the explanted blade revealed a blunted and missing tip (Figure 3A). Guided by the knowledge that a fragment was unaccounted for, the neurosurgical team engaged in careful exploration of the surgical field. The team located the broken metallic tip embedded near the inner wall of the frontal sinus and the adjacent damaged frontal lobe and successfully retrieved it from the intracranial compartment (Figure 3B). Following the removal of all foreign bodies and complete debridement of necrotic brain tissue and bone shards, extensive reconstructive efforts were required. Cranialization of the frontal sinus was thoroughly performed. This involved completely exenterating the remaining sinus mucosa and meticulously sealing the nasofrontal tracts and bony defects using bone wax, fibrin glue, and a TachoSil patch. Finally, a vascularized pericranial flap was utilized to achieve a watertight dural repair, thereby isolating the sterile intracranial space from the nasal cavity. Intraoperative navigation, fluoroscopy, and postoperative endoscopic evaluations were not required, as the extensive craniotomy provided sufficient direct macroscopic and microscopic visualization.

The patient was subsequently transferred to the neurosurgical intensive care unit for close observation. Postoperatively, cerebrospinal fluid (CSF) leakage was noted on day 3, indicating a challenge to the initial dural seal. Concurrent with the CSF leak, signs of infection emerged. Wound and CSF cultures were immediately collected, and empiric broad-spectrum intravenous antibiotics were initiated on the same day. The wound cultures subsequently yielded *Serratia odorifera* and coagulase-negative *Staphylococci* (CoNS). Under the guidance of the Infectious Disease department, intravenous cefepime was administered based on the confirmed susceptibility results for *Serratia odorifera*, while intravenous vancomycin was utilized empirically to cover the CoNS. This tailored antibiotic regimen was maintained for a total treatment duration of 2 weeks. Fortunately, the CSF leakage and superficial infection resolved conservatively with the targeted medical therapy and bed rest, without the need for reoperation or a CSF diversion procedure (such as lumbar drainage).

The patient’s inflammatory markers showed a dramatic and positive response to the targeted medical therapy. The serum C-reactive protein (CRP) levels dropped significantly from 16.9 mg/dL (measured on postoperative day 1) to 0.97 mg/dL (measured on postoperative day 11). During this period, no clinical signs suggestive of infection, such as fever or wound discharge, were observed. A postoperative follow-up CT scan was performed, which confirmed the gross total removal of the foreign body, proper cranialization of the frontal sinus, and the absence of any significant delayed intracranial hematoma or abscess (Figure 3C).

Postoperatively, the patient’s neurological status gradually improved, reaching a drowsy state (GCS E3V4M6) on postoperative day (POD) 7. By POD 14, he became fully alert and was safely transferred to a regional rehabilitation hospital, achieving a Glasgow Outcome Scale (GOS) score of 5 and a modified Rankin Scale (mRS) score of 1. At the 1-month postoperative outpatient follow-up, the patient maintained an alert mental status with no focal neurological deficits, signs of wound infection, or CSF leakage, maintaining a GOS of 5 and an mRS of 1. Subsequently, formal outpatient visits to our neurosurgical department were discontinued as the patient was admitted to a dedicated psychiatric hospital for long-term psychiatric care. Consequently, no long-term postoperative neuroimaging (such as CT, MRI, or delayed DSA) was obtained after discharge. However, through telephonic follow-up with the patient’s mother (his primary guardian), it was confirmed that the patient remains hospitalized for ongoing psychiatric management without any neurological deficits, surgical site complications, or recurrent infections.

## 3. Discussion

pTBI represents a devastating and highly complex subset of head trauma, historically associated with exceptionally high mortality rates and profound long-term neurological morbidity. The acute management paradigms and surgical algorithms for pTBI are heavily dictated by the exact injury mechanism (missile versus non-missile), the specific anatomical structures violated by the traumatic tract, and the promptness of medical and surgical intervention. Our case illustrates a remarkably rare mechanism of self-inflicted pTBI involving a trans-frontal sinus trajectory executed with a kitchen knife in a patient suffering from chronic schizophrenia. This combination of severe physical trauma and complex psychiatric comorbidity demands a highly nuanced, multidisciplinary approach spanning emergency medicine, radiology, neurosurgery, infectious disease, and psychiatry.

Although a trans-frontal sinus trajectory is rare, it presents significant clinical challenges that directly affect patient morbidity and mortality. Unlike low-velocity penetrations that strictly breach the intact calvarium, a breach of the frontal sinus introduces unique pathophysiological challenges. This specific anatomical route creates a direct, persistent communication among the external environment, the colonized paranasal cavities, and the sterile intracranial compartment. Consequently, this mechanism significantly elevates the immediate and delayed risk of life-threatening complications such as CSF rhinorrhea, fulminant bacterial meningitis, and delayed brain abscess formation. Furthermore, the close anatomical proximity of the frontal sinus and the anterior cranial fossa floor poses a severe and unique threat to the major vessels of the anterior cerebral vasculature.

In current neurotrauma practice, clinicians can often underestimate the true extent of deep intracranial damage based on the initial clinical presentation. Recent authoritative guidelines and multicenter studies emphasize that cerebrovascular injuries, including traumatic intracranial aneurysms, vessel transections, and arteriovenous fistulas, are common after penetrating head trauma [1,2]. According to current pTBI imaging algorithms, CTA is recommended as the initial non-invasive screening modality; however, conventional DSA is strongly indicated if the traumatic tract approaches major intracranial vessels or if initial CTA is non-diagnostic [1,2]. In our patient, although CTA was performed upon admission, the kitchen knife generated massive metallic streak and beam-hardening artifacts, rendering precise evaluation of the adjacent anterior cerebral vasculature impossible. Therefore, despite its inherent procedural risks, conventional DSA proved highly useful in this specific case prior to foreign body extraction, and it may be considered when initial CTA is non-diagnostic due to severe metallic artifacts. It provided high-resolution, artifact-free, dynamic visualization to help rule out traumatic pseudoaneurysms, vascular lacerations, or active extravasation. Ultimately, confirming the absence of major vascular injury on DSA was a highly favorable prognostic factor that significantly altered the surgical risk profile in our case.

The surgical strategy in the context of non-missile pTBI extends far beyond the simple, mechanical extraction of the foreign body. Our case prominently highlights the essential need for the operating surgeon to carefully inspect the extracted object for structural integrity immediately upon removal. In our case, the prompt identification and extraction of the retained fragment from the deep frontal parenchyma were successfully performed. However, the management of retained fragments requires a careful risk–benefit analysis. While retained fragments in situ are associated with a risk of deep migration, initiating additional local brain injury, or serving as an intractable nidus for delayed abscess formation, the decision to extract them should depend on factors such as fragment composition, location, accessibility, neurovascular risk, and contamination, weighed against the potential for iatrogenic injury. To safely achieve these surgical goals in this specific case, we deliberately performed a wide bifrontal craniotomy. This extensive exposure was necessary to ensure the highly controlled extraction of the blade under direct microscopic visualization, avoiding a deep penetrating trajectory that might exacerbate vascular injury while concurrently repairing the frontal sinus [3]. The utilization of a wide bifrontal craniotomy, rather than a limited local craniectomy, was a deliberate strategic choice to minimize blind retraction injury. In trans-frontal sinus penetrations, the penetrating trajectory typically disrupts multiple contiguous anatomical compartments. Generous surgical exposure provides an expansive view of the anterior skull base, facilitating the direct identification of associated cribriform plate fractures or dural lacerations that require primary repair. Furthermore, this wide visualization is indispensable for identifying hidden bony fragments or small metallic splinters that might otherwise be overlooked within a restricted operative field. Retaining these micro-fragments undetected poses a significant risk of delayed mechanical parenchymal shearing from brain pulsation and serves as a persistent nidus for chronic infection. Consequently, maximizing the initial surgical exposure is crucial to ensure macroscopically complete debridement and to facilitate effective reconstruction of the anterior skull base. Crucially, to definitively prevent ongoing CSF leakage and block the pathway for ascending bacterial infection, rigorous cranialization of the frontal sinus was performed utilizing a highly vascularized, autologous pericranial flap.

The postoperative medical management of trans-frontal sinus pTBI is heavily focused on effectively controlling the inevitable polymicrobial contamination resulting from the sinus violation. In our specific case, the subsequent microbiological cultures definitively confirmed the presence of *Serratia odorifera* and coagulase-negative *Staphylococci*. As noted in the case description, these isolated organisms represented a localized, superficial wound infection with CSF contamination, rather than fulminant meningitis or a deep intracranial abscess. This localized infection was effectively managed with a targeted intravenous regimen of cefepime and vancomycin. In particular, *Serratia odorifera* is a rare opportunistic pathogen in neurosurgical site infections. Its isolation in this patient underscores the vulnerability of the sterile intracranial compartment to uncommon environmental flora introduced via the paranasal sinuses. Effective eradication of this organism using vancomycin and cefepime highlights the importance of immediate empiric broad-spectrum coverage, followed by prompt de-escalation to culture-directed targeted therapy in trans-sinus neurotrauma. This strategy aligns with multicenter reviews of pTBI confirming that infection rates can be significant, reinforcing the absolute necessity of combining aggressive surgical debridement with such tailored antimicrobial regimens in cases with sinus breach [4]. Additionally, neuro-intensive care management must anticipate neurological sequelae beyond infection. In this case, levetiracetam was administered as seizure prophylaxis for an intended duration of 1 month. It was initially administered intravenously and later transitioned to oral formulation. Importantly, no clinical seizures were observed during the patient’s entire hospital course. This specific regimen aligns with current management guidelines for penetrating neurotrauma, which recommend early prophylaxis to mitigate the well-recognized risk of post-traumatic epilepsy associated with dural violation and cortical damage [2,5].

A particularly noteworthy aspect of this case is the favorable neurological recovery achieved by the patient, despite his initially severe presentation characterized by a stuporous mental state and extensive physical trauma. This highly positive outcome directly challenges the traditional “therapeutic nihilism” that has often historically shadowed the management of severe pTBI cases. Recent updated guidelines emphasize that low initial Glasgow Coma Scale (GCS) scores should not inevitably preclude aggressive surgical and medical intervention, as a significant subset of patients can achieve functional independence [5]. This modern paradigm is particularly relevant in the specific context of psychiatric patients presenting with self-inflicted injuries, where the initial neurological exam may be heavily confounded by psychotropic medications, agitation, or underlying mental status rather than irreversible structural brain damage alone [6]. Furthermore, the perioperative management of patients with chronic schizophrenia necessitates close multidisciplinary coordination with psychiatric services to safely reintroduce and titrate atypical antipsychotic regimens within the neuro-intensive care unit. Preventing acute psychotic relapse or severe postoperative agitation is important, as uncooperative behavior can compromise surgical wound integrity, lead to the accidental removal of medical devices, and severely complicate serial neurological assessments during the critical recovery phase.

## 4. Limitations

This study has limitations. First, as a single retrospective case report, these findings have limited generalizability. Second, long-term in-person neurosurgical follow-up was limited due to the patient’s transfer to an outside psychiatric facility, necessitating reliance on telephonic confirmation via his guardian. This highlights the real-world challenge of maintaining long-term outpatient follow-up in patients with severe schizophrenia.

## 5. Conclusions

In summary, while this single retrospective case report has limited generalizability, it illustrates a possible multidisciplinary approach to managing a severe, anatomically complex self-inflicted pTBI involving trans-frontal sinus penetration. Rather than prescribing a rigid algorithm, this case highlights the critical importance of individualized vascular assessment—such as considering DSA when CT is compromised by metallic artifacts. Furthermore, it emphasizes the value of controlled extraction of foreign bodies and retained fragments, rigorous skull-base repair via frontal sinus cranialization, vigilant infectious surveillance with culture-directed targeted antibiotics, and comprehensive psychiatric follow-up. Adhering to these principles through a tailored, multidisciplinary strategy can help improve survival and functional outcomes in similarly complex presentations [7].

## Data Availability

The original contributions presented in this study are included in the article. Further inquiries can be directed to the corresponding author.

## Abbreviations

The following abbreviations are used in this manuscript:

CRP	C-reactive protein
CSF	Cerebrospinal fluid
CT	Computed tomography
DSA	Digital subtraction angiography
GCS	Glasgow Coma Scale
pTBI	Penetrating traumatic brain injury
